# 12‐month outcomes of ranibizumab versus aflibercept for macular oedema in central retinal vein occlusion: data from the FRB! registry

**DOI:** 10.1111/aos.15014

**Published:** 2021-09-13

**Authors:** Mateusz Niedzwiecki, Adrian Hunt, Vuong Nguyen, Hemal Mehta, Catherine Creuzot‐Garcher, Pierre‐Henry Gabrielle, Martin Guillemin, Samantha Fraser‐Bell, Jennifer Arnold, Ian L. McAllister, Mark Gillies, Daniel Barthelmes

**Affiliations:** ^1^ Department of Ophthalmology University Hospital Zurich & University of Zurich Zurich Switzerland; ^2^ Department of Ophthalmology Westmead Hospital Westmead NSW Australia; ^3^ The Save Sight Institute Sydney Medical School The University of Sydney Sydney NSW Australia; ^4^ Ophthalmology Department Royal Free London NHS Foundation Trust London UK; ^5^ Department of Ophthalmology Dijon University Hospital Dijon France; ^6^ Marsden Eye Specialists Parramatta NSW Australia; ^7^ Centre for Ophthalmology and Visual Science Lions Eye Institute The University of Western Australia Perth WA Australia

**Keywords:** aflibercept, CRVO, cystoid, macula, oedema, ranibizumab

## Abstract

**Purpose:**

To compare 12‐month treatment outcomes of eyes receiving aflibercept or ranibizumab for macular oedema secondary to central retinal vein occlusion (CRVO) in routine clinical practice.

**Methods:**

296 treatment‐naïve eyes receiving either aflibercept (171 eyes, 2 mg) or ranibizumab (125 eyes, 0.5 mg) for macular oedema secondary to CRVO were recruited retrospectively from centres using the prospectively designed FRB! registry. The primary outcome measure was the mean change in LogMAR letter scores of visual acuity (VA). Secondary outcomes included change in central subfield thickness (CST), injections and visits, time to first grading of inactivity, switching and non‐completion from baseline to 12 months.

**Results:**

Baseline VA (SD) was somewhat better in aflibercept‐ versus ranibizumab‐treated eyes (42.5 ± 25.5 letters versus 36.9 ± 26 letters; p = 0.07) with similar CST (614 (240) μm versus 616 (234) μm: p = 0.95). The 12‐month adjusted mean (95%CI) VA change was +16.6 (12.9, 20.4) letters for aflibercept versus +9.8 (5.5, 14.1) letters for ranibizumab (p = 0.001). The mean (95%CI) adjusted change in CST was significantly greater in aflibercept‐ versus ranibizumab‐treated eyes: −304 (−276, −333) µm versus −252 (−220, −282) µm (p < 0.001). Both groups had a median (Q1, Q3) of 7 (5, 9) injections and 10 (8,13) visits. Aflibercept‐treated eyes became inactive sooner than ranibizumab (p = 0.02). Switching occurred more commonly from ranibizumab (26 eyes, 21%) than from aflibercept (9 eyes, 5%) (p < 0.001).

**Conclusion:**

Both aflibercept and ranibizumab improved VA and reduced CST in eyes with CRVO in routine clinical practice, with aflibercept showing significantly greater improvements in this comparative analysis.

## Introduction

Treatment of central retinal vein occlusion (CRVO) has progressed from prevention of sight‐threatening sequelae (Hayreh 2003) to vascular endothelial growth factor (VEGF) inhibitors, which randomized controlled trials (RCTs) suggest can improved vision significantly (Campochiaro et al. 2011; Boyer et al. 2012; Brown et al. 2013; Korobelnik et al. 2014). There are, however, limited data showing that these impressive RCT outcomes are being achieved in routine clinical care and whether the licenced drugs, aflibercept and ranibizumab, are equivalent in the general population.

Randomized controlled trials (RCTs) mandate frequent intravitreal injections that pose a significant treatment burden which is difficult to always achieve in routine clinical practice (Kiss et al. 2014; Lotery & Regnier 2015; Stallworth et al. 2020). Various retrospective observational analyses suggest that fewer injections are given in the first 12 months than in RCTs, with correspondingly lower visual acuity gains (Chatziralli et al. 2017, 2018; Kitagawa et al. 2018; Callizo et al. 2019). On average, 4–5 injections were given in the first 12 months, resulting in an average visual gain of approximately 1.2 lines (Lotery & Regnier 2015; Gale et al. 2020; Stallworth et al. 2020).

The LEAVO study was a randomized clinical trial that reported that ranibizumab was non‐inferior to aflibercept in CRVO (Hykin et al. 2019). There were selected cohorts treated under controlled conditions following a strict induction protocol followed by a PRN regimen from week 16 to week 96, which may be similar to routine clinical care. The VA outcomes at 12 months were similar between aflibercept‐ and ranibizumab‐treated eyes (Hykin et al. 2019). The SCORE2 study reported that bevacizumab was ‘non‐inferior’ to aflibercept in a heterogeneous group of eyes with CRVO or HRVO (Scott et al. 2017).

The quality of data from routine clinical practice is variable. ‘Mining’ large data sets from electronic medical records currently produces lower quality data, such as a recent report using data from the US Retina database, where baseline visual acuity could not be identified in 130 25 of 301 06 (35%) of eyes receiving anti‐VEGF treatment for age‐related macular degeneration (Kiss et al. 2020). Outcomes registries with prespecified mandatory fields – such as the Fight Retinal Blindness! Project – require users to enter all data within prespecified ranges for the visit to be ‘finalized’ and accepted into the database. Finalization rates consistently exceed 95% of recorded visits. The additional effort users make produces higher quality, complete data sets.

Here, we report a comparative analysis of 12‐month treatment outcomes of a large cohort of patients in routine clinical practice who received aflibercept or ranibizumab for macular oedema secondary to CRVO from participating centres in the Fight Retinal Blindness! Project.

## Materials and Methods

### Design and setting

We conducted a retrospective analysis of eyes with CRVO treated with approved intravitreal anti‐VEGF agents. Treatment was tracked in routine clinical practice within the prospectively designed retinal vein occlusion module of the Fight Retinal Blindness! Registry (Gillies et al. 2014). Participants were treatment‐naïve and managed at clinics in Australia, France, Switzerland and the United Kingdom. Ethics and data protection approval was obtained from the University of Sydney and the Royal Australian and New Zealand College of Ophthalmologists (HREC#16.09), the French Institutional Review Board (2017_CLER‐IRB_ll‐05), the Cantonal Ethics Commission in Zurich (PB_2016‐00264) and the Caldicott Guardian of the Royal Free London NHS Foundation Trust (Dr Kilian Hynes). The study adhered to the STROBE checklists for reporting observational studies (von Elm et al. 2008) and followed the tenets of the Declaration of Helsinki. All patients gave informed consent. An ‘opt‐in’ informed consent was sought from patients from France, Switzerland and the United Kingdom. An ‘opt‐out’ patient consent was approved by Ethics committees in Australia.

### Data sources and measurements

Data were collected at each clinical visit including the number of letters read on a logarithm of the minimum angle of resolution (logMAR) VA Chart (highest of uncorrected, corrected or pinhole), the activity (presence of intraretinal cystoid changes) of cystoid macular oedema (CME [yes/no]), the central subfield thickness (CST [µm]) measured using spectral‐domain optical coherence tomography (OCT), treatment given, other ocular procedures and ocular adverse events. Relevant systemic risk factors or ocular conditions were recorded at baseline only, as was the type of RVO (CRVO, hemi‐RVO or branch‐RVO) (McAllister et al. 2014), and if a fluorescein angiogram was performed, whether macular or peripheral ischaemia was documented. Drug choice and treatment frequency were at the physician’s discretion in consultation with the patient reflecting routine clinical practice.

### Patient selection

Treatment‐naïve eyes that started treatment with either ranibizumab (0.5 mg Lucentis, Genentech Inc/Novartis) or aflibercept (2 mg Eylea, Bayer) from 1 June 2014 to 1 June 2019 were studied. Eyes with hemi‐RVO or branch‐RVO were excluded. Eyes that had at least three visits and were followed for 12 months were defined as ‘completers’. Switchers were defined as eyes that received ≥2 injections of the other drug prior to switching. Visits occurring after the switch were not included in this analysis. Eyes that did not complete 12 months of observations were defined as ‘non‐completers’.

### Outcomes

The main outcome was the mean change in VA at 12 months between anti‐VEGF agents. Secondary outcomes were the mean change in CST, number of visits and the number of injections. Other event‐based outcomes of interest were first grading of CME inactivity, switching and non‐completion rates over 12 months.

### Statistical analysis

Descriptive data were summarized using the mean, standard deviation, median, first and third quartiles, and percentages where appropriate. Eyes were observed from the first treatment visit to their 12‐month (365 ± 30 days) visit. T‐tests, Wilcoxon signed‐rank tests, chi‐square tests and Fisher’s exact tests were used as appropriate to compare baseline characteristics between ranibizumab‐ and aflibercept‐treated eyes. Calculation of crude visual and anatomic outcomes at 12 months used the last observation carried forward (LOCF) for switchers and non‐completers. We used longitudinal generalized additive mixed‐effects models to compare VA and CST outcomes between the treatments over the 12‐month period with the interaction between injection group and time as the main predictor. The longitudinal models included all visits up until 12 months from completers, non‐completers and switchers without imputation of missing data (i.e. LOCF). Visits occurring after an eye switched drugs were not included. We adjusted for age and baseline VA or CST as fixed effects, and nesting of outcomes within doctor and patient (for bilateral cases) as random effects. We used predictions from these models to plot predicted VA and CST, and the difference in the mean predicted VA and CST, over 12 months for each drug.

Generalized Poisson linear mixed models were used to compare visits and injections with an offset for log days of follow‐up. Kaplan–Meier survival analysis was used to assess the time to first grading of CMO inactivity, non‐completion and switching. A Cox‐proportional hazards model was used to compare time to inactivity between treatment groups. Generalized Poisson and Cox‐proportional hazards models were adjusted for age, baseline VA and baseline CST as fixed effects, and nesting of outcomes within doctor and patient as random effects.

All analyses were conducted using r version 4.0.0 (http://www.R‐project.org/) using the *glmmTMB* (V1.0.1) package for generalized linear mixed‐effects regression, the *mgcv* (V1.8‐31) package for generalized additive mixed models and the *coxme* (V2.2‐16) and *survival* (V3.1‐12) packages for time‐to‐event analyses (R Core Team 2020).

## Results

### Study participants

We identified 296 treatment‐naïve patient eyes (125 ranibizumab and 171 aflibercept) in 291 patients with cystoid macular oedema secondary to CRVO that started treatment with either ranibizumab or aflibercept from 1 June 2014 to 1 June 2019 (Table 1).

**Table 1:** Demographic characteristics of all treatment‐naïve CRVO eyes commencing ranibizumab or aflibercept treatment 2014–2019.

	Overall	Ranibizumab	Aflibercept	p‐value
Eyes, *n*	296	125	171	
Patients, *n*	291	122	170	
Female, %	47%	47%	47%	1.00
Age, mean (SD)	72 (13)	73 (12)	71 (13)	0.14
VA, mean (SD)	40.1 (25.8)	36.9 (26)	42.5 (25.5)	0.07
≥70 letters, %	13%	13%	13%	1.00
≤35 letters, %	41%	45%	38%	0.29
FFA Performed, *n* (%)*	176 (59%)	75 (60%)	101 (59%)	0.96
Macular Ischaemia, *n* (%)	20 (7%)	9 (7%)	11 (6%)	0.81
Peripheral Ischaemia, *n* (%)	75 (25%)	37 (30%)	38 (22%)	0.16
CST, mean (SD)	615 (236)	614 (240)	616 (234)	0.96
Hypertension, %	60%	58%	61%	0.80
Glaucoma, %	16%	14%	17%	0.53
Country, %				
Australia	29%	30%	29%	
France	31%	39%	25%	
Switzerland	24%	15%	32%	
United Kingdom	15%	16%	15%	

n = number, SD = standard deviation, VA = visual acuity (logMAR letters), FFA = fundus fluorescein angiography, CST = central subfield thickness (in microns).

* Not mandatorily performed or documented.

There were no statistically significant differences at baseline in eyes grouped by VEGF inhibitor. Eyes receiving ranibizumab were slightly older (73 versus 71 years; p = 0.14), had lower mean baseline visual acuity (36.9 versus 42.5 letters; p = 0.07), more presented with VA ≤ 35 letters (45% versus 38%, p = 0.29) and were less likely to have a history of systemic hypertension and glaucoma than those receiving aflibercept. The groups had very similar mean (SD) baseline CST (ranibizumab 614 (240) μm versus aflibercept 616 (234) μm; p = 0.95). Fundus fluorescein angiography (FFA) was performed in 60% of all eyes studied. Twenty eyes overall that had documented macular ischaemia were more likely to have baseline visual acuity ≤35 letters (p = 0.01); however, the treatment groups had no significant difference in documented ischaemia at baseline including both macular (7%) and peripheral ischaemia (25%).

### Visual outcomes at 12 months

Mean crude VA improvement (95% confidence interval [CI]) was higher for aflibercept than for ranibizumab (+13.1 letters [9.4, 16.8] versus +9.9 [5.8, 14.1] (p = 0.26), including eyes that switched or dropped out (using LOCF) (Table 2).

**Table 2:** 12‐month outcomes in all eyes and stratified by anti‐VEGF agent received. Significant p‐values comparing ranibizumab and aflibercept are highlighted in bold.

	Overall	Ranibizumab	Aflibercept	p‐value
No of Eyes	296	125	171	
Baseline VA, mean (SD)	40.1 (25.8)	36.9 (26)	42.5 (25.5)	0.07
Final VA, mean (SD)	51.9 (28.5)	46.9 (29.4)	55.5 (27.3)	**0.01**
Crude VA change, mean (95% CI)	11.8 (9, 14.5)	9.9 (5.8, 14.1)	13.1 (9.4, 16.8)	0.26
Adjusted VA change, mean (95% CI)^†^		9.8 (5.5, 14.1)	16.6 (12.9, 20.4)	**0.001**
Gained ≥ 15 letters (%)	46%	40%	50%	0.10
Lost ≥ 15 letters (%)	10%	10%	11%	0.95
VA ≥ 70%Baseline / %Final	13% / 37%	13% / 30%	13% / 42%	1.00/0.05
VA ≤ 35%Baseline / %Final	41% / 28%	45% / 34%	38% / 24%	0.29/0.09
CST Baseline, mean (SD)	615 (236)	614 (240)	616 (234)	0.95
CST Final, mean (SD)	336 (169)	369 (179)	314 (159)	**0.01**
CST Change, mean (95% CI)	‐279 (−311, −247)	‐245 (−292, −197)	‐302 (−345, −258)	0.10
Adjusted CST Change, mean (95% CI)^†^		‐252 (−220, −282)	‐304 (−276, −333)	**<0.001**
Completers, *n* (%)	236 (80%)	99 (79%)	137 (80%)	0.70
Switchers, *n* (%)	35 (12%)	26 (21%)	9 (5%)	**<0.001**
Lost to follow‐up, *n* (%)	60 (20%)	26 (21%)	34 (20%)	
Injections, median (Q1, Q3)*	7 (5, 9)	6 (4, 9)	8 (5, 9)	0.62
Visits, median (Q1, Q3)*	10 (8, 13)	10 (7, 13)	10 (8, 13)	0.84

n = number, VA = visual acuity, SD = standard deviation, CI = confidence interval, CST = central subfield thickness, Q1 = first quartile, Q3 = third quartile.

All eyes – includes completers, switchers and non‐completers. ‘Completers’ – eyes with 12 months of observation from the start of treatment, ‘switchers’ – eyes receiving ≥2 injections of the other treatment drug prior to completion of 12 months from the start of treatment. Observations were included in the analysis only up to the first occurrence of switching agents. ‘Non‐Completers’ – eyes not completing 12 months of observations from the start of treatment.

* Last observation carried forward for switchers and non‐completers.

^†^ Calculated from longitudinal models adjusting for age and baseline VA (fixed effects), and practice and intra‐patient correlation for bilateral cases (random effects).

This trend was more pronounced in eyes presenting with baseline VA ≤ 35 letters (38% in the aflibercept‐treated group and 45% in the ranibizumab‐treated group) with mean crude VA improvement in the aflibercept group of +24.6 (18.5, 30.7) letters versus +16.6 (10.4, 22.8) letters in the ranibizumab group (p = 0.07) from similar mean baseline VA: 13.7 (13.7) letters versus 11.9 (13.2) letters (p = 0.46) (Table S1). The treatment groups started with very similar proportions of eyes with VA ≥ 70 at baseline (13%); however, more eyes in the aflibercept group (42%) had VA ≥ 70 letters at 12 months than in the ranibizumab group (30%; p = 0.05).

**Fig. 1:**
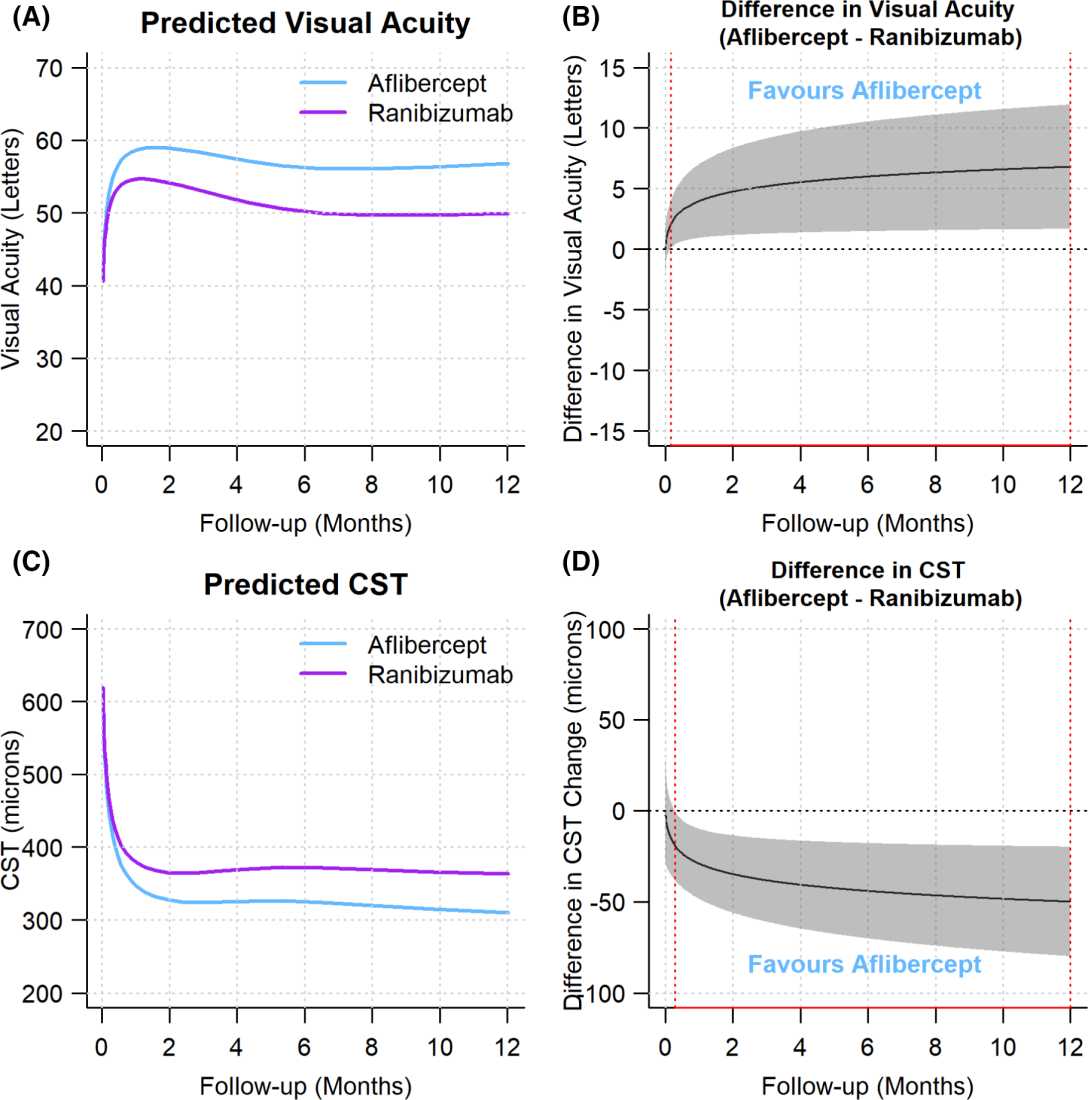
Graphical representation of vision and CST by drug. Predictions from longitudinal generalized additive models of adjusted visual acuity (A, B) and CST (C, D). Red dotted lines in (B, D) indicate periods in which the confidence interval of the difference between drugs no longer crosses zero.

The generalized additive mixed model (*Methods*) predicted a mean adjusted VA change (95% CI) that was greater with aflibercept +16.6 (12.9, 20.4) letters than +9.8 (5.5, 14.1) letters with ranibizumab group (p = 0.001). The mean adjusted VA over 12 months for each group is shown in Fig. 1A, while Fig. 1B shows the difference in longitudinal trend between drugs. Eyes on aflibercept achieved larger gains in VA than ranibizumab which are statistically significant from the first week onwards to 12 months.

### Macular thickness

Both drugs were effective in reducing macular thickness (Table 2). Mean baseline CST (SD) was very similar (ranibizumab 614 (240) μm versus aflibercept 616 (234) μm; p = 0.95); however, at 12 months, the mean CST (SD) was significantly lower in the aflibercept group at 313 (157) μm versus 370 (180) μm in the ranibizumab group (p = 0.01). The difference in crude effect on CST of aflibercept compared with ranibizumab was more marked in the 121 eyes (41%) presenting with poor VA ≤ 35 letters (Table S1). This subset presented with similar mean CST of 716 (286) μm in the aflibercept group versus 693 (256) μm in the ranibizumab group (p = 0.67); however, the aflibercept‐treated eyes had lower final CST of 296 (145) μm versus 388 (218) μm (p = 0.03) and greater crude CST change of −419 (−498, −341) μm versus −305 (−389, −221) μm (p = 0.08), than the ranibizumab‐treated eyes at 12 months.

Application of a generalized additive mixed model predicted a greater mean adjusted CST change (95% CI) for aflibercept of −304 (−276, −333) μm vs. −252 (−220, −282) for ranibizumab (p < 0.001). The statistically significant longitudinal trend favouring aflibercept is shown in Fig. 1C,D extending from the first 2 weeks through 12 months.

### Treatments and visits

The completers (80%) in the aflibercept group had a median (Q1, Q3) of 8 (5, 9) injections, and 10 (8, 13) visits, while the completers (79%) in the ranibizumab group had 6 (4, 9) injections and 10 (7, 14) visits (p = 0.62, 0.84; Table 2). Thus, aflibercept‐treated eyes received somewhat more injections, but this difference was not statistically significant. The range in injections delivered was from 1 to 13 over 12 months. Both groups received a similar number of injections: completers had a mean total of 7.4 injections (7.5 aflibercept, 7.2 ranibizumab) over 12 months. The mean number of injections in the first 6 months was 4.8 (4.8 aflibercept, 4.7 ranibizumab), and 2.6 (2.7 aflibercept, 2.6 ranibizumab) in the second 6 months. The median time between each of the 1st to 5th injections was 4, 4, 6 and 6 weeks. Twenty‐nine eyes received fewer than 4 injections, and in 12 of these, the final was VA <20 letters; however, in the other 17 eyes, the median final VA was 76 (55, 80) letters at 12 months. Cataract surgery was performed in 9 ranibizumab‐treated eyes and 4 aflibercept eyes with YAG capsulotomy performed in one eye from each group.

### Inactivity, switching and loss to follow‐up

**Fig. 2:**
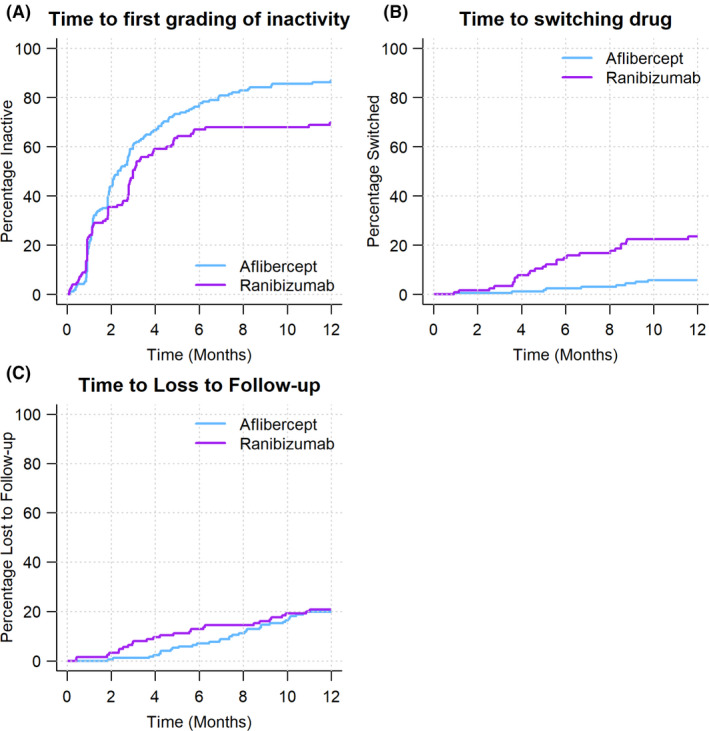
Kaplan–Meier curve for first grading of inactivity, time to switching and dropout by drug.

Kaplan–Meier survival analysis was used to compare ranibizumab and aflibercept in terms of time to first grading of inactivity, switching and loss to follow‐up (Fig. 2). Inactivity was recorded at least once in 12 months in 227 eyes (96% of completers), with the first occurrence at a median (Q1, Q3) of 58 (29, 98) days. The Cox‐proportional hazards model predicted aflibercept achieved inactivity sooner than ranibizumab (p = 0.02).

Thirty‐five eyes (12%) switched treatment within 12 months, more commonly from ranibizumab (26 eyes, 21%) than from aflibercept (9 eyes, 5%) (p < 0.001) (Table 2). The median (Q1, Q3) time to switching for all eyes combined was 155 days (112, 252). Eyes switched from ranibizumab to a dexamethasone implant (6 eyes), to aflibercept (17 eyes) or to bevacizumab (2 eyes) with a median VA of 59 (36, 65) letters at the time of switch. Eyes switched from aflibercept with a lower median VA of 45 (29, 50) letters at the time of switch to a dexamethasone implant (5 eyes) or ranibizumab (4 eyes).

Sixty eyes (20%) dropped out before 12 months. The non‐completion rate was similar in the ranibizumab group (21%) and the aflibercept group (20%). The overall median (Q1, Q3) time to dropout was 193 days (119, 271). Documented reasons for loss to follow‐up included 2 deaths, a medical contraindication in 1 patient, futility of treatment in 3 eyes, 7 patients declined further treatment while 10 patients went to another doctor.

### Adverse events

Macular changes affecting vision were newly observed during follow‐up in 28 eyes (ERM, macular hole, pigment clumping, atrophy) with a mean (SD) baseline VA of 15 (20) letters and mean 12‐month VA of 22 (28) letters. Neovascular complications in either the anterior segment (16 eyes) or posterior segment (17 eyes) led to poor outcomes with a combined mean (SD) VA of 13 (21) letters at 12 months. Eighty‐three eyes received panretinal photocoagulation with a 12‐month mean VA (SD) of 36 (30) letters from a baseline VA of 26 (28.5) letters. Eyes receiving PRP (83 eyes) had fewer injections (SD) with 6.4 (3.4) compared to 7.3 (3) in eyes that did not receive PRP (p = 0.04). Vitreous haemorrhage was reported in 13 eyes that received a mean (SD) of 3.8 (2.7) injections. Significantly fewer injections 2.5 (1.6) were given to 16 eyes that developed rubeotic glaucoma compared to the rest of the cohort (p < 0.001). Rubeotic glaucoma developed more often in ranibizumab‐treated eyes (12 eyes vs 4 aflibercept‐treated eyes; p = 0.01); however, these eyes received fewer injections 1.8 injections vs. 4.25 injections respectively. Injection numbers overall, irrespective of the agent, were strongly associated with rubeotic glaucoma occurrence (p < 0.001) suggesting the injection number rather than the drug was associated with rubeotic glaucoma. There was one retinal detachment with VA at 12 months of light perception but no reported cases of endophthalmitis or traumatic cataract following 1915 injections.

## Discussion

We report significant improvements in VA and reductions in macular thickness in eyes receiving aflibercept or ranibizumab treatment for CRVO in routine clinical practice. Both groups were well‐matched for gender, age, visual acuity and CST at baseline. Both groups had similar numbers of visits and injections during the 12‐month period. Our comparative analysis found that eyes receiving aflibercept had greater visual gains and reductions in CST.

Significant differences in the molecular structure and mode of action of the drugs we studied may be the reason for the better outcomes we found with aflibercept for CRVO. While ranibizumab is a humanized monoclonal antibody, aflibercept acts as a decoy‐receptor for VEGF and may offer superior VEGF suppression due to higher binding affinity against VEGF (Papadopoulos et al. 2012) as well as longer intravitreal half‐life (Stewart & Rosenfeld 2008). This may be particularly important in eyes with CRVO, which have very high vitreous levels of VEGF (Aiello et al. 1994).

While treatment is mandated in RCTs, treatment patterns greatly differ in routine clinical practice due to various factors, including patient compliance, cost and individual re‐treatment preferences. As a consequence, the number of injections is often lower than in RCTs as observed in the current analysis and other database studies (Lotery & Regnier 2015). Many analyses of outcomes from routine clinical practice have reported 4–5 injections for CRVO in the first year, in contrast to RCTs which gave on average 8.8–9.6 aflibercept injections (Campochiaro et al. 2011) or 8.4 ranibizumab injections (Brown et al. 2013; Korobelnik et al. 2014) within the first 12 months. Centres participating in the current analysis gave more injections than have previously been reported from routine clinical practice (a median of 7 for both aflibercept and ranibizumab), which is only slightly fewer than in RCTs.

The combination of stronger and potentially longer VEGF suppression of aflibercept may be one of the main drivers for better clinical outcomes since the more prolonged suppression may compensate for the somewhat lower number of injections. Cystoid macula oedema secondary to CRVO may be a particularly attractive indication for new longer acting anti‐VEGF agents.

### Patient population

The patient population in this analysis from routine clinical practice was older (mean 72 years) than patients included in RCTs using aflibercept or ranibizumab (range 61.5–69.7 years) (Campochiaro et al. 2011; Brown et al. 2013; Korobelnik et al. 2014; Larsen et al. 2018). Patient eyes in the current analysis had worse average baseline VA scores (40.1 letters) than those included in RCTs (range 47.4–53 letters), with less thickened mean baseline CST of 615 µm (range in RCTs 665–693 µm) (Campochiaro et al. 2011; Brown et al. 2013; Korobelnik et al. 2014; Larsen et al. 2018).

### Visual outcomes and macular thickness

Visual outcomes for aflibercept and ranibizumab, both adjusted (16.6 and 9.8) and unadjusted (13.1 and 9.9), from this analysis were slightly inferior to those observed in RCTs (13.9 to 18.9 letters; Campochiaro et al. 2011; Brown et al. 2013; Korobelnik et al. 2014; Scott et al. 2017). Lower gains in vision observed in this study were likely due to differences in baseline characteristics and lack of mandated treatment every 4 weeks in the first 6 months. Also, the time from the occurrence of the CRVO to treatment initiation was not limited as in RCTs. Fundus fluorescein angiography, performed in around 60% of eyes, was evenly distributed between both treatment groups. A total of 7% and 25% of eyes showed signs of macular ischaemia and peripheral ischaemia respectively. It seems unlikely that eyes with macular ischaemia contributed significantly to the observed reduced VA gains of the total cohort, since previous reports in ranibizumab‐treated eyes found that macular ischaemia did not influence VA outcomes (Larsen et al. 2016; Tadayoni et al. 2017).

Aflibercept‐treated eyes had more significant reductions in CST than ranibizumab‐treated eyes. The Cox‐proportional hazards model predicted that aflibercept was significantly faster in achieving CMO inactivity than ranibizumab (p = 0.02).

### Switching treatments and loss of follow‐up

Switching occurred in around 12% of eyes, mainly from ranibizumab (21%) rather than from aflibercept (5%). The reason for switching was not recorded. We hypothesize that it might have included a perceived lack of response by the treating physician. Loss of follow‐up was observed in 20% of eyes, which is comparable to other observational studies. Ranibizumab was approved for the treatment of CME secondary to CRVO much earlier than aflibercept. This might have influenced the decision to switch too.

### Adverse events

The rate and nature of adverse events, such as macular atrophy, pigment clumping or epiretinal membrane, in our study population was relatively low and about the same as in other diseases treated with intravitreal anti‐VEGF such as diabetic retinopathy or age‐related macular degeneration.

An important detail is the number of lasers and the fact that, despite anti‐VEGF treatment, rubeotic glaucoma developed in 16 eyes – those eyes had significantly fewer injections than the rest of the cohort (mean 2.5 [1.6]). It has now been established that the requirement for panretinal laser photocoagulation in proliferative diabetic retinopathy can be reduced by anti‐VEGF therapy (Writing Committee for the Diabetic Retinopathy Clinical Research et al. 2015; Sivaprasad et al. 2017). With the relatively higher VEGF levels in patients with CRVO, one might expect a similar benefit from anti‐VEGF therapy (Aiello et al. 1994). However, the evidence base is not as clear for the risk of neovascularization in eyes with CRVO receiving anti‐VEGF therapy, especially when treatment is stopped. Data from routine clinical practice may provide useful insights to the development and management of rubeosis in eyes receiving VEGF inhibitors for CRVO.

### Strengths and weaknesses

The current analysis has limitations that are inherent to studies using data from routine clinical practice. In contrast to RCTs, treatment decisions are based on the physician’s observation in consultation with the patient. The choice of when to treat and to schedule the next appointment also relies on the patient’s availability to integrate frequent appointments into a busy work scheduled. Normally, no reading centre recommendations or protocols are followed as is the case in RCTs. There was no randomization to treatment groups, which, while not significant, resulted in some differences in baseline characteristics. We accounted for this partially by adjusting for baseline factors that might impact the outcome, such as age, VA and CST.

The strengths of the current study are the large sample size and an adequate representation of how anti‐VEGF drugs are used in routine clinical practice in a number of centres that treat CRVO. The present study, which had fortuitously well‐matched baseline characteristics, is unlikely to overestimate either of the drugs' effectiveness (Concato et al. 2000).

Observational studies may suffer from poor data quality. For example, baseline and 12‐month visual acuity values could only be identified in around half of the 30 000 otherwise eligible eyes with neovascular age‐related macular degeneration in a recent analysis from the IRIS database (Kiss et al. 2020). By contrast, the FRB! database only accepts ‘finalized’ data which is 100% complete and within prespecified ranges, the finalization rate is consistently above 95%.

## Conclusions

This study found that both aflibercept and ranibizumab improved VA and reduced macular thickness over 12 months in eyes with CRVO. Aflibercept led to significantly greater improvements, both in VA and CST. Longer‐term observational studies are warranted to verify whether the initial benefit of aflibercept and ranibizumab is maintained.

## Supporting information


**Table S1**. 12 Month outcomes in CRVO eyes with Baseline VA ≤35 Letters and VA >35 Letters and stratified by Anti‐VEGF agent received.

## References

[aos15014-bib-0001] Aiello LP , Avery RL , Arrigg PG et al. (1994): Vascular endothelial growth factor in ocular fluid of patients with diabetic retinopathy and other retinal disorders. N Engl J Med 331: 1480–1487. 7526212 10.1056/NEJM199412013312203

[aos15014-bib-0002] Boyer D , Heier J , Brown DM et al. (2012): Vascular endothelial growth factor Trap‐Eye for macular edema secondary to central retinal vein occlusion: six‐month results of the phase 3 COPERNICUS study. Ophthalmology 119: 1024–1032. 22440275 10.1016/j.ophtha.2012.01.042

[aos15014-bib-0003] Brown DM , Heier JS , Clark WL et al. (2013): Intravitreal aflibercept injection for macular edema secondary to central retinal vein occlusion: 1‐year results from the phase 3 COPERNICUS study. Am J Ophthalmol 155: 429–437. 23218699 10.1016/j.ajo.2012.09.026

[aos15014-bib-0004] Callizo J , Ziemssen F , Bertelmann T et al. (2019): Real‐world data: ranibizumab treatment for retinal vein occlusion in the OCEAN study. Clin Ophthalmol 13: 2167–2179. 31806930 10.2147/OPTH.S209253 PMC6847987

[aos15014-bib-0005] Campochiaro PA , Brown DM , Awh CC , Lee SY , Gray S , Saroj N , Murahashi WY & Rubio RG (2011): Sustained benefits from ranibizumab for macular edema following central retinal vein occlusion: twelve‐month outcomes of a phase III study. Ophthalmology 118: 2041–2049. 21715011 10.1016/j.ophtha.2011.02.038

[aos15014-bib-0006] Chatziralli I , Theodossiadis G , Chatzirallis A , Parikakis E , Mitropoulos P & Theodossiadis P (2018): RANIBIZUMAB FOR RETINAL VEIN OCCLUSION: predictive factors and long‐term outcomes in real‐life data. Retina 38: 559–568. 28248827 10.1097/IAE.0000000000001579

[aos15014-bib-0007] Chatziralli I , Theodossiadis G , Moschos MM , Mitropoulos P & Theodossiadis P . (2017): Ranibizumab versus aflibercept for macular edema due to central retinal vein occlusion: 18‐month results in real‐life data. Graefe's Arch Clin Exp Ophthalmol 255: 1093–1100. 28214955 10.1007/s00417-017-3613-1

[aos15014-bib-0008] Concato J , Shah N & Horwitz RI (2000): Randomized, controlled trials, observational studies, and the hierarchy of research designs. N Engl J Med 342: 1887–1892. 10861325 10.1056/NEJM200006223422507 PMC1557642

[aos15014-bib-0009] Gale R , Gill C , Pikoula M et al. (2020): Multicentre study of 4626 patients assesses the effectiveness, safety and burden of two categories of treatments for central retinal vein occlusion: intravitreal anti‐vascular endothelial growth factor injections and intravitreal Ozurdex injections. Br J Ophthalmol [Epub ahead of print]. 10.1136/bjophthalmol-2020-317306. PMC8140590 32962992

[aos15014-bib-0010] Gillies MC , Walton R , Liong J et al. (2014): Efficient capture of high‐quality data on outcomes of treatment for macular diseases: the fight retinal blindness! Project. Retina 34: 188–195. 23836194 10.1097/IAE.0b013e318296b271

[aos15014-bib-0011] Hayreh SS (2003): Management of central retinal vein occlusion. Ophthalmologica 217: 167–188. 12660480 10.1159/000068980

[aos15014-bib-0012] Hykin P , Prevost AT , Vasconcelos JC et al. (2019): Clinical effectiveness of intravitreal therapy with Ranibizumab vs Aflibercept vs bevacizumab for macular edema secondary to central retinal vein occlusion: a randomized clinical trial. JAMA Ophthalmol 137: 1256. 31465100 10.1001/jamaophthalmol.2019.3305 PMC6865295

[aos15014-bib-0013] Kiss S , Campbell J , Almony A , Shih V , Serbin M , LaPrise A & Wykoff CC (2020): Management and outcomes for neovascular age‐related macular degeneration: analysis of United States electronic health records. Ophthalmology 127: 1179–1188. 32345477 10.1016/j.ophtha.2020.02.027

[aos15014-bib-0014] Kiss S , Liu Y , Brown J , Holekamp NM , Almony A , Campbell J & Kowalski JW (2014): Clinical utilization of anti‐vascular endothelial growth‐factor agents and patient monitoring in retinal vein occlusion and diabetic macular edema. Clin Ophthalmol 8: 1611–1621. 25210429 10.2147/OPTH.S60893 PMC4155807

[aos15014-bib-0015] Kitagawa S , Yasuda S , Ito Y , Ueno S , Iwase T & Terasaki H (2018): Better prognosis for eyes with preserved foveal depression after intravitreal ranibizumab injection for macular edema secondary to central retinal vein occlusion. Retina 38: 1354–1360. 28538263 10.1097/IAE.0000000000001707

[aos15014-bib-0016] Korobelnik JF , Holz FG , Roider J et al. (2014): Intravitreal aflibercept injection for macular edema resulting from central retinal vein occlusion: one‐year results of the phase 3 GALILEO Study. Ophthalmology 121: 202–208. 24084497 10.1016/j.ophtha.2013.08.012

[aos15014-bib-0017] Larsen M , Waldstein SM & Boscia F et al. (2016): Individualized ranibizumab regimen driven by stabilization criteria for central retinal vein occlusion: twelve‐month results of the CRYSTAL study. Ophthalmology 123: 1101–1111. 26896124 10.1016/j.ophtha.2016.01.011

[aos15014-bib-0018] Larsen M , Waldstein SM , Priglinger S et al. (2018): Sustained benefits from ranibizumab for central retinal vein occlusion with macular edema: 24‐month results of the CRYSTAL study. Ophthalmol Retina 2: 134–142. 31047340 10.1016/j.oret.2017.05.016

[aos15014-bib-0019] Lotery A & Regnier S (2015): Patterns of ranibizumab and aflibercept treatment of central retinal vein occlusion in routine clinical practice in the USA. Eye 29: 380–387. 10.1038/eye.2014.308. 25572584 PMC4366471

[aos15014-bib-0021] McAllister IL , Tan MH , Smithies LA & Wong WL (2014): The effect of central retinal venous pressure in patients with central retinal vein occlusion and a high mean area of nonperfusion. Ophthalmology 121: 2228–2236. 25037752 10.1016/j.ophtha.2014.05.031

[aos15014-bib-0022] Papadopoulos N , Martin J , Ruan Q et al. (2012): Binding and neutralization of vascular endothelial growth factor (VEGF) and related ligands by VEGF Trap, ranibizumab and bevacizumab. Angiogenesis 15: 171–185. 22302382 10.1007/s10456-011-9249-6 PMC3338918

[aos15014-bib-0023] R Core Team (2020): R: a language and environment for statistical computing. Vienna, Austria: R Foundation for Statistical Computing. Available at: https://www.R‐project.org/.

[aos15014-bib-0024] Scott IU , VanVeldhuisen PC , Ip MS et al. (2017): Effect of bevacizumab vs aflibercept on visual acuity among patients with macular edema due to central retinal vein occlusion: The SCORE2 randomized clinical trial. JAMA 317: 2072–2087. 28492910 10.1001/jama.2017.4568 PMC5710547

[aos15014-bib-0025] Sivaprasad S , Prevost AT , Vasconcelos JC et al. (2017): Clinical efficacy of intravitreal aflibercept versus panretinal photocoagulation for best corrected visual acuity in patients with proliferative diabetic retinopathy at 52 weeks (CLARITY): a multicentre, single‐blinded, randomised, controlled, phase 2b, non‐inferiority trial. Lancet 389: 2193–2203. 28494920 10.1016/S0140-6736(17)31193-5

[aos15014-bib-0026] Stallworth JY , Akshay ST , Constantine R , Stinnett SS & Fekrat S (2020): Treatment patterns and clinical outcomes for central retinal vein occlusion in the antivascular endothelial growth factor era. J VitreoRetinal Dis 4(1): 13–21. 10.1177/2474126419878922 PMC9976087 37009559

[aos15014-bib-0027] Stewart MW & Rosenfeld PJ (2008): Predicted biological activity of intravitreal VEGF Trap. Br J Ophthalmol 92: 667–668. 18356264 10.1136/bjo.2007.134874

[aos15014-bib-0028] Tadayoni R , Waldstein SM , Boscia F et al. (2017): Sustained benefits of Ranibizumab with or without laser in branch retinal vein occlusion: 24‐month results of the BRIGHTER study. Ophthalmology 124: 1778–1787. 28807635 10.1016/j.ophtha.2017.06.027

[aos15014-bib-0031] von Elm E , Altman DG , Egger M , Pocock SJ , Gøtzsche PC & Vandenbroucke JP (2008): The strengthening the reporting of observational studies in epidemiology (STROBE) statement: guidelines for reporting observational studies. J Clin Epidemiol 61: 344–349. 18313558 10.1016/j.jclinepi.2007.11.008

[aos15014-bib-0033] Writing Committee for the Diabetic Retinopathy Clinical Research N , Gross JG , Glassman AR et al. (2015): Panretinal photocoagulation vs intravitreous ranibizumab for proliferative diabetic retinopathy: a randomized clinical trial. J Am Med Assoc 314: 2137–2146. 10.1001/jama.2015.15217 PMC5567801 26565927

