# Supplementary material for: 12‐month outcomes of ranibizumab versus aflibercept for macular oedema in central retinal vein occlusion: data from the FRB! registry
Source: Acta Ophthalmol. 2021 Sep 13;100(4):e920–7. doi: 10.1111/aos.15014 (PMC9292733; doi:10.1111/aos.15014)
Supplement: Supplementary file 1 — Table S1. 12 Month outcomes in CRVO eyes with Baseline VA ≤35 Letters and VA >35 Letters and stratified by Anti‐VEGF agent received. [file AOS-100-e920-s001.docx]

**Supplementary Table S1**. 12 Month outcomes in CRVO eyes with Baseline VA ≤35 Letters and VA >35 Letters and stratified by Anti-VEGF agent received

|  | **Baseline VA ≤35 Letters** | | **Baseline VA >35 Letters** | |
| --- | --- | --- | --- | --- |
|  | **Ranibizumab** | **Aflibercept** | **Ranibizumab** | **Aflibercept** |
| No of Eyes | 56 | 65 | 69 | 106 |
| Baseline VA, mean (SD) | 11.9 (13.2) | 13.7 (13.7) | 57.3 (12.6) | 60.1 (10.6) |
| Final VA, mean (SD) | 28.4 (27.6) | 38.3 (27.5) | 61.8 (21.3) | 66.1 (21.2) |
| Crude VA change, mean (95% CI) | 16.6 (10.4, 22.8) | 24.6 (18.5, 30.7) | 4.6 (-0.7, 9.8) | 6.0 (1.8, 10.2) |
| Gained ≥ 15 letters (%) | 45% | 62% | 36% | 43% |
| Lost ≥ 15 letters (%) | 2% | 3% | 16% | 15% |
| VA ≥ 70 %Baseline / %Final | 0% / 9% | 0% / 14% | 23% / 48% | 21% / 59% |
| VA ≤ 35 %Baseline / %Final | 100% / 62% | 100% / 45% | 0% / 10% | 0% / 11% |
| CST Baseline, mean (SD) | 693 (256) | 716 (286) | 563 (216) | 557 (174) |
| CST Final, mean (SD) | 388 (218) | 296 (145) | 357 (148) | 325 (167) |
| CST Change, mean (95% CI) | -305 (-389, -221) | -419 (-498, -341) | -205 (-259, -151) | -232 (-278, -186) |
| Completers, n (%) | 42 (75%) | 49 (75%) | 57 (83%) | 88 (83%) |
| Switchers, n (%) | 14 (25%) | 3 (5%) | 12 (17%) | 6 (6%) |
| Lost to follow up, n (%) | 14 (25%) | 16 (25%) | 12 (17%) | 18 (17%) |
| Injections, median (Q1, Q3) * | 6 (4, 9) | 8 (5, 9) | 7 (4, 9) | 7 (5, 9) |
| Visits, median (Q1, Q3) * | 10 (7, 14) | 11 (9, 14) | 10 (8, 12) | 10 (8, 12) |

*Injections and visits were calculated for completers only
